# Myxomatous Mysteries: Decoding the Oral Focal Mucinosis

**DOI:** 10.7759/cureus.67921

**Published:** 2024-08-27

**Authors:** Saptarshi Das, Arunit Chatterjee, Rudra Prasad Chatterjee, Sangeeta Sinha, Mehebuba Sultana, Sk Abdul Mahmud, Neha Shah, Mousumi Pal

**Affiliations:** 1 Oral and Maxillofacial Pathology, Guru Nanak Institute of Dental Sciences and Research, Kolkata, IND; 2 Oral and Maxillofacial Pathology, Guru Nanak Institute of Dental Sciences of Research, Kolkata, IND

**Keywords:** hyaluronidase, fibroblast, periodic acid-schiff (pas), s-100, silver reticulin, myxomatous lesion, mucicarmine, hyaluronic acid, alcian blue, focal mucinosis

## Abstract

Oral focal mucinosis (OFM) is a unique benign lesion of the oral cavity with uncertain etiology which is analogous to cutaneous focal mucinosis. It mainly affects women in their fourth and fifth decades of life. The diagnosis of this condition is based on histopathological examination, as it lacks characteristic clinical and radiographic features. Its pathophysiology is associated with fibroblasts producing excessive amounts of hyaluronic acid, which causes localized myxomatous changes. Here, we describe the occurrence of this rare entity in a 54-year-old female patient involving attached gingiva of the left posterior mandibular region along with emphasis on its histopathological and histochemical findings to differentiate it from clinically and microscopically look-alike lesions.

## Introduction

Oral focal mucinosis (OFM) is an unfamiliar benign tumor with uncertain origin [1]. It is most common in the fourth and fifth decades of life, while it has also been documented in children and teenagers on rare occasions [2]. It is more frequent in women than in men, having a ratio of 2.1:1 [3]. The pathogenesis of OFM is unknown, however, the lesion is thought to be caused by the local buildup of mucin in connective tissue due to increased hyaluronic acid synthesis by fibroblasts [4]. It preferentially affects keratinized mucosa like the hard palate and gingiva [5]. Typically, the lesion appears as a nodule that is painless, sessile, or pedunculated, with a size ranging from a few millimeters to 5 cm in diameter and exhibits the same color as that of the normal surrounding mucosa. The surface is usually smooth and nonulcerated, while some cases have a lobulated appearance [1,6]. Histologically, OFM is characterized by a well-defined myxomatous area containing mucinous material and is encircled by dense collagenous connective tissue stroma. Since the lesion is benign in nature, surgical excision is the treatment of choice [7-11].

## Case presentation

**Figure 1:**
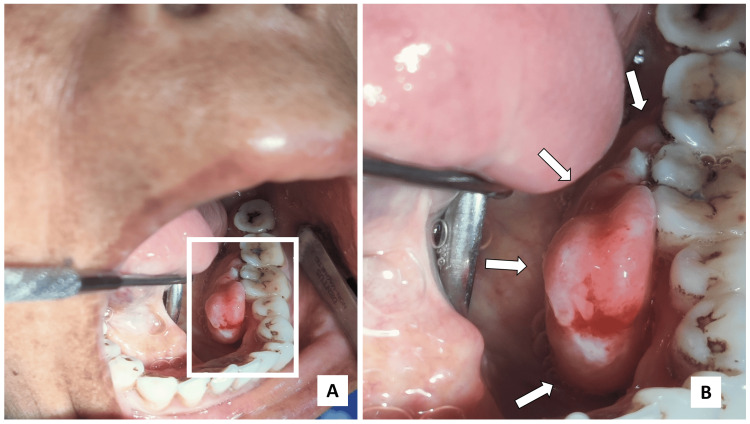
Intraoral examination (A) Lobulated, pedunculated mass over the lingual aspect of gingival region w.r.t. 34,35,36,37; (B) Anteroposterior extent of the lesion was from the distal aspect of 34 to mesial aspect of 37 while superoinferiorly it extended from the occlusal surface of regional teeth to depth of the lingual sulcus. White arrows depict the extent of the growth.

A 54-year-old female reported to the outpatient department (OPD) with the chief complaint of growth over the left lower back teeth region for the last 6 months. No history of previous trauma was noted. On intraoral examination, the lesion was round to ovoid, sessile, smooth surfaced, lobulated mass approximately measuring 2.5 × 2 × 1.5 cm in diameter over the left mandibular attached gingiva with respect to the lingual aspect of the first premolar to the second molar region (Figure 1A).

**Figure 2:**
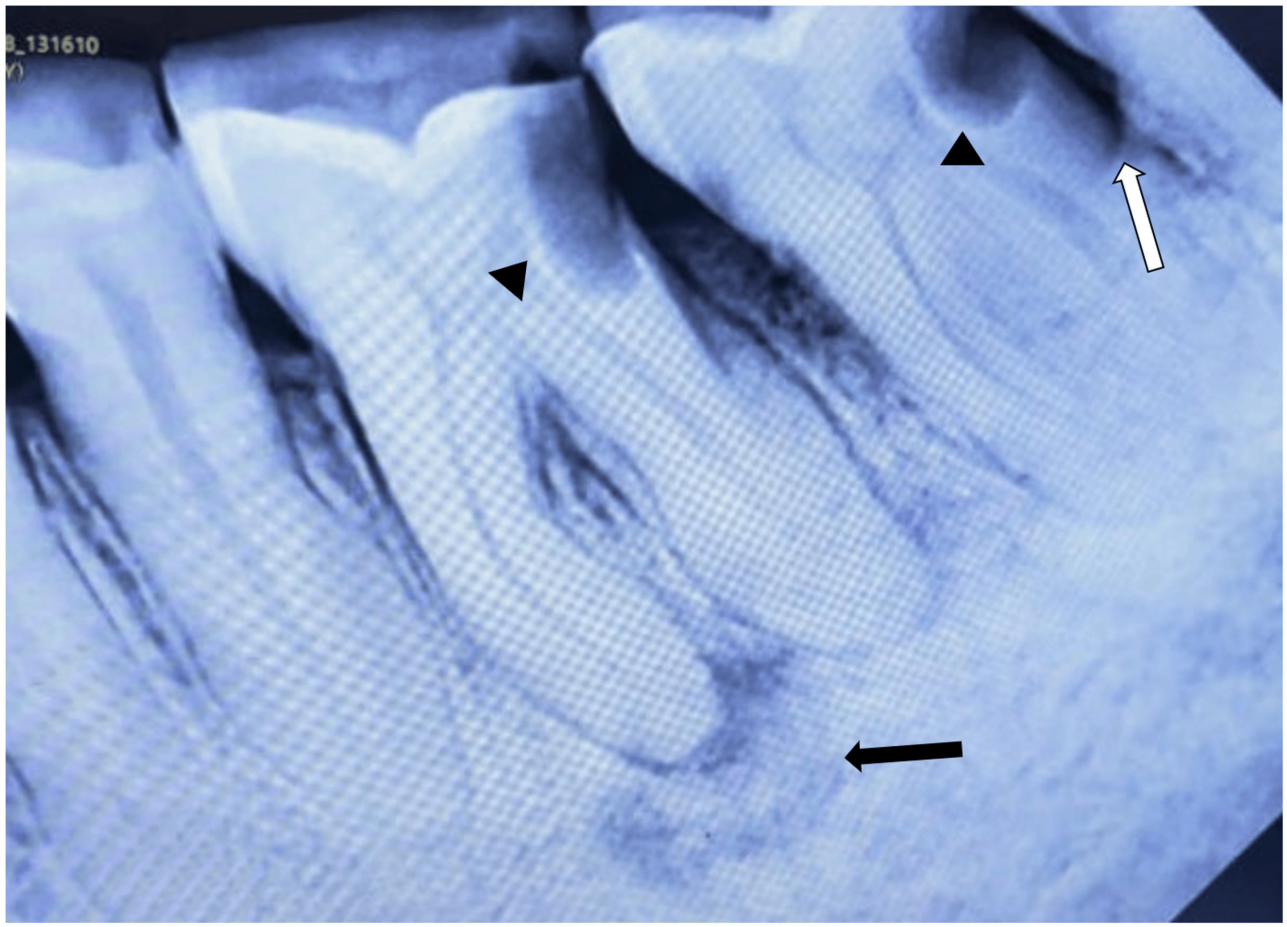
Radiographic findings Proximal carious lesion w.r.t. 36 and 37 (arrowheads) along with widened PDL space. Angular bone loss was noted in the interproximal region of 37 and 38 (white arrow). Black arrow showing periapical radiolucency w.r.t. mesial root of 36.

The lesion extended anteriorly from the distal surface of 34 to the mesial surface of 37 posteriorly. Superiorly, it extended up to the occlusal surface of posterior teeth and inferiorly extended up to the depth of the lingual sulcus (Figure 1B). The lesion was soft to firm in consistency and was non-tender on palpation. There was no significant radiographic finding related to the lesion. Regional teeth showed periodontal ligament (PDL) space widening due to progression of bacterial infection from proximal caries along with discontinuous lamina dura and irregular periapical radiolucency w.r.t. mesial root of 36. Angular bone loss in the interproximal region of 37 and 38 was noted suggestive of poor periodontal condition (Figure 2).

**Figure 3:**
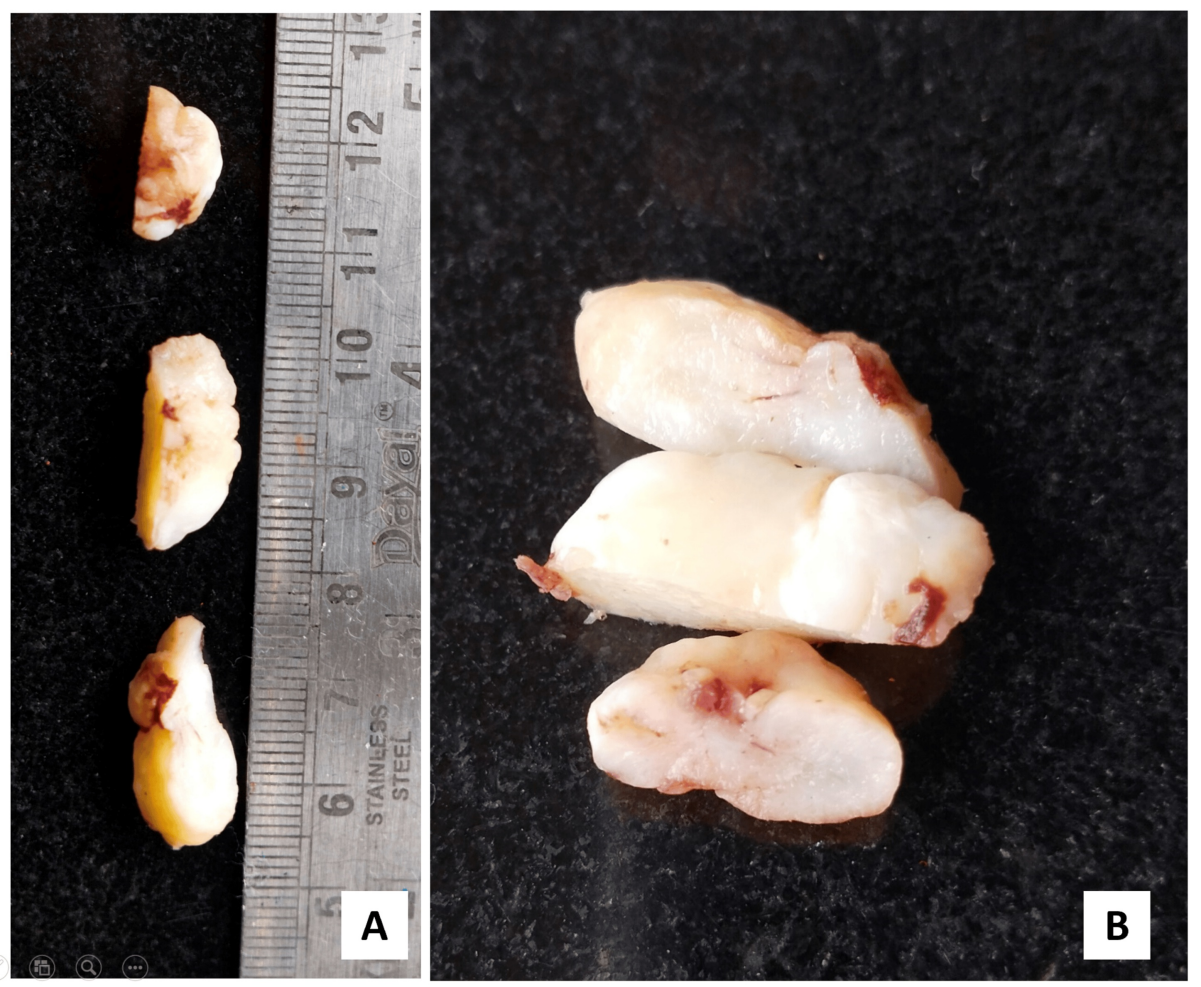
Gross examination of the mass (A) A well-circumscribed lobulated mass measuring 2.5 × 2 × 1.5 cm; (B) cut surface revealed glistening white appearance.

The provisional diagnosis of a fibromatous lesion was made and an excisional biopsy was performed as a curative measure. A gross examination of the mass revealed a well-circumscribed glistening white cut surface (Figure 3).

**Figure 4:**
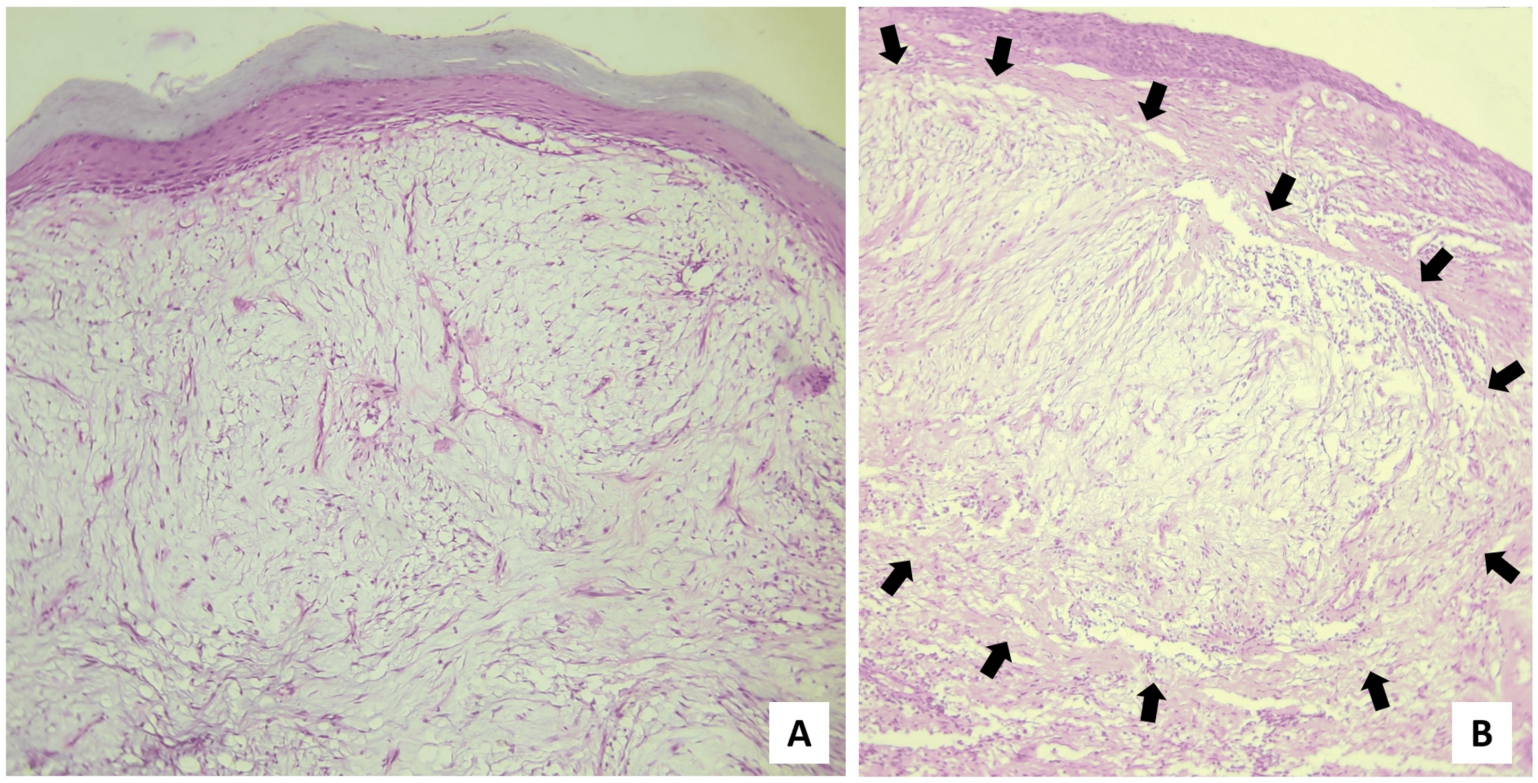
Scanner view (×40 magnification) of the H&E-stained sections (A) Hyperparakeratinized, atrophic, stratified, squamous surface epithelium supported by myxomatous connective tissue stroma, (B) myxomatous area surrounded by dense fibrovascular connective tissue stroma. The black arrows demarcate the border between the two aforementioned areas.

On microscopical examination, the H&E-stained sections revealed the presence of hyperparakeratinized, stratified squamous surface epithelium which was atrophic in nature, with focal ulceration and relatively flattened rete ridges, supported by a myxomatous connective tissue stroma having widely dispersed collagen. The presence of endothelial-lined blood vessels, a few of which revealed perivascular infiltration of chronic inflammatory cells was noted in connective tissue areas. In some instances, the myxomatous area was surrounded by dense, normal connective tissue (Figure 4). 

**Figure 5:**
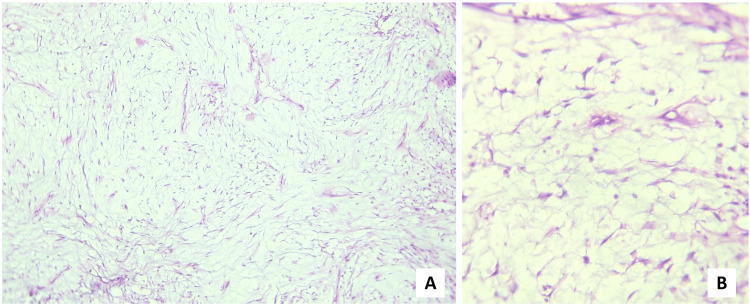
H&E-stained sections Fusiform and stellate-shaped fibroblasts having fibrillar processes along with widely dispersed collagen ((A) ×100 & (B) ×400 magnification).

The fibroblasts within the mucinous area were fusiform and stellate-shaped, demonstrating delicate, fibrillar processes (Figure 5).

**Figure 6:**
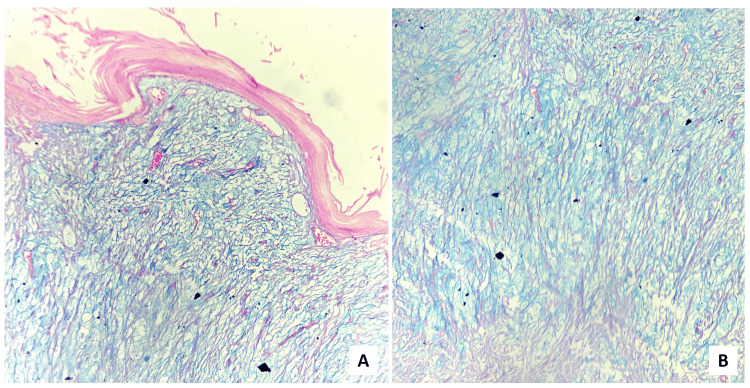
(A) Scanner (×40 magnification) & (B) high-power (×400 magnification) view depicting Alcian Blue positive myxomatous stroma

**Figure 7:**
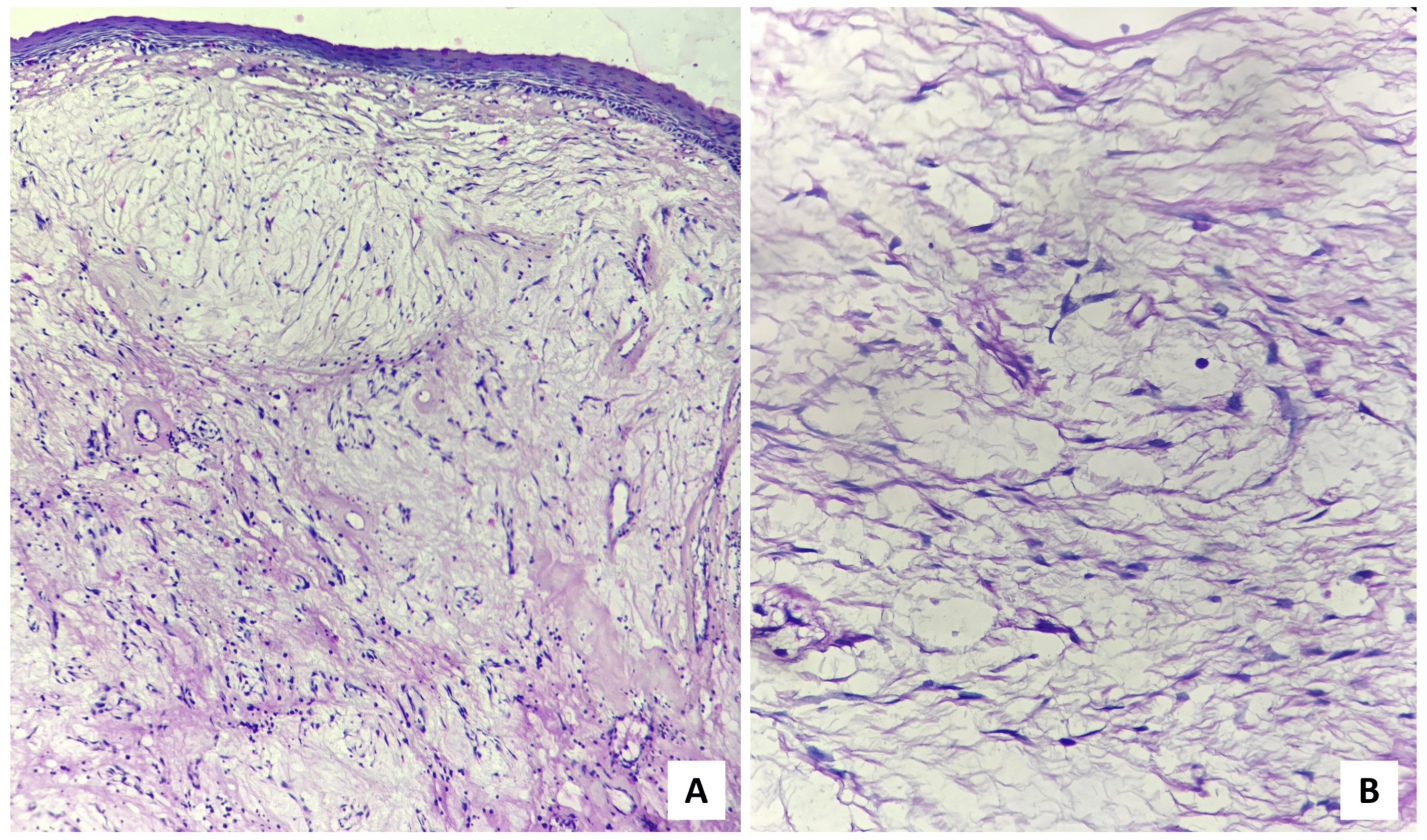
The myxomatous stroma was negative for PAS staining ((A) ×40 and (B) ×400 magnification) PAS: Periodic Acid Schiff

**Figure 8:**
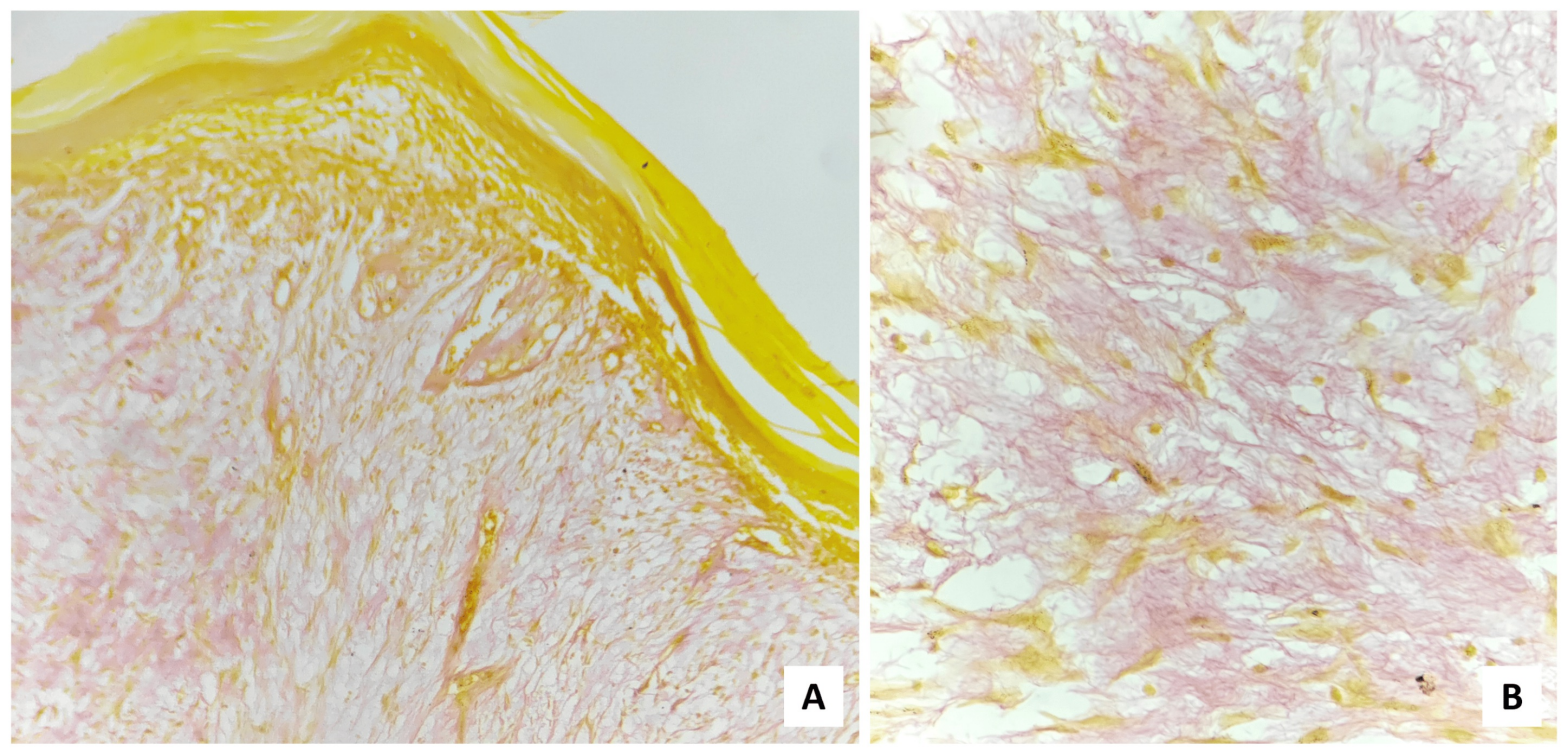
Mucicarmine stain demonstrated weak positivity ((A) ×40 and (B) ×400 magnification)

Few capillaries were seen within the lesion. No significant inflammatory cells were noted. The histopathological diagnosis of OFM was made. To confirm the diagnosis, staining using Alcian Blue (Figure 6), Periodic Acid Schiff (PAS) (Figure 7), and mucicarmine (Figure 8) were performed to demonstrate the nature of the mucin. Positive Alcian Blue while negative PAS test revealed it to be acid mucin, while weak positivity of mucicarmine suggested non-epithelial origin of the mucin.

**Figure 9:**
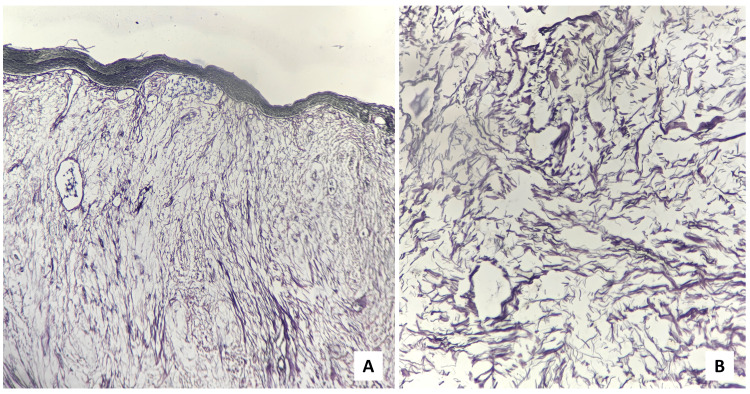
(A) Scanner (×40 magnification) & (B) high-power (×400 magnification) view showing negativity for silver reticulin stain

Silver reticulin (Figure 9) stain was also done to demonstrate the reticulin fibers, which was negative.

**Figure 10:**
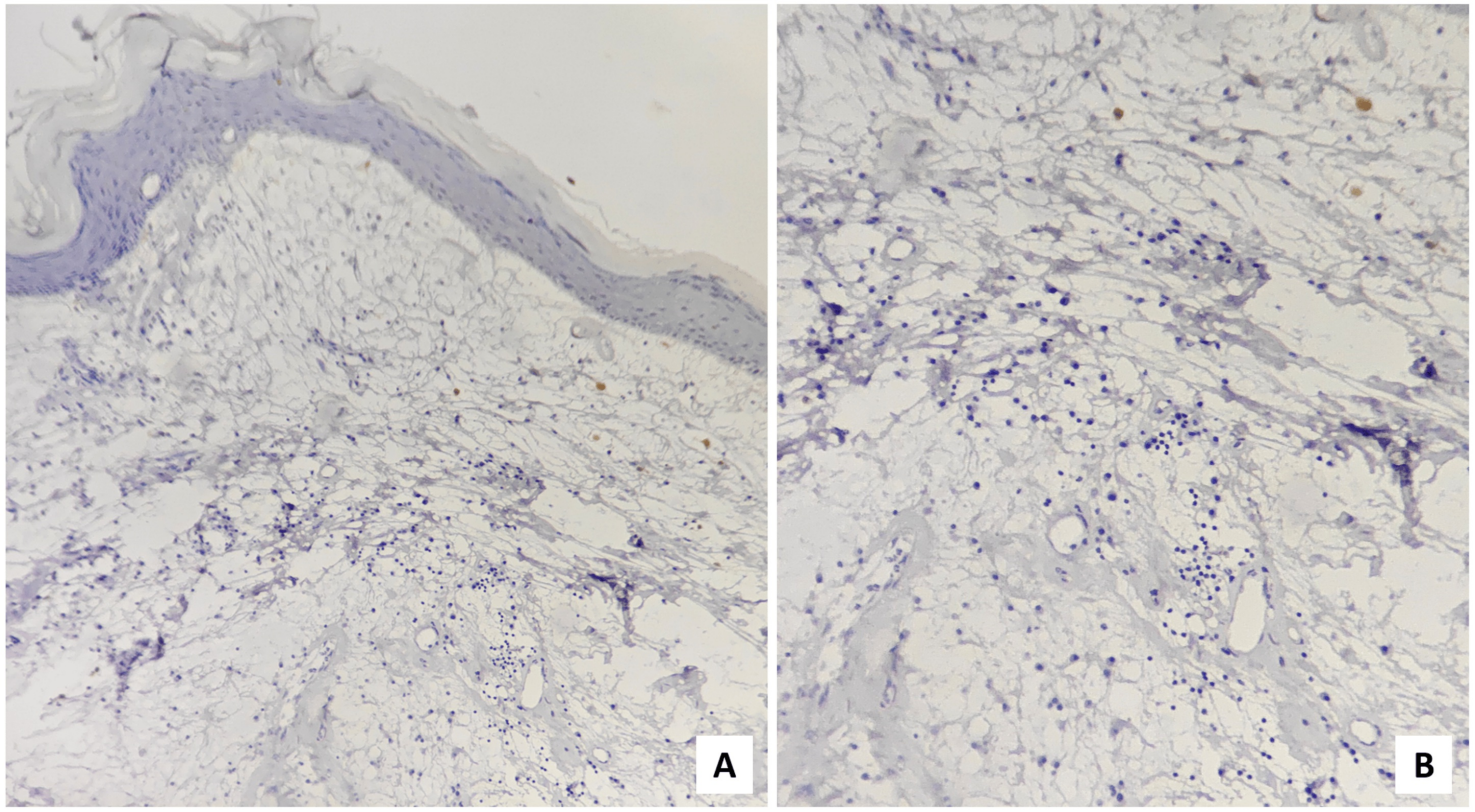
(A) Scanner (×40 magnification) & (B) low-power (×100 magnification) view of immunohistochemical staining exhibited S-100 negative myxomatous area.

S-100 (Figure 10) was performed to differentiate the lesion from nerve sheath myxoma, which was also negative.

On the basis of clinical, histopathological, and histochemical findings (Table 1), the final diagnosis of OFM was reached.

**Table 1:** Summary of the different histochemical methods performed to exclude other myxomatous lesions

Histochemical Staining	Result	Inference	Lesions Excluded
Alcian Blue (Figure 6)	Positive	The mucoid substance was acidic in nature.	Mucocele
PAS (Figure 7)	Negative	No neutral mucin was present	
Mucicarmine (Figure 8)	Weakly positive	The mucin was non-epithelial in origin.	
Silver reticulin (Figure 9)	Negative	Absence of reticular fibers	Soft-tissue myxoma, fibroma with myxomatous changes, neurofibroma with myxomatous changes
S-100 (Figure 10)	Negative	No neural component present	Nerve sheath myxoma, neurofibroma

## Discussion

The term “Cutaneous Focal Mucinosis” refers to a dome-shaped, asymptomatic nodule, often found on the face and trunk that was first identified by Johnson and Helwig in 1966 [5]. Tomich (1974) described the oral counterpart of this lesion [12] and concluded that majority of lesions that are identified as oral soft-tissue myxomas are actually this type of lesions [12]. Based on clinical, histological, and histochemical data, the mucosal lesions are thought to be the oral counterparts of the cutaneous myxoid cyst or cutaneous focal mucinosis with changes explained only by variations in their anatomic location [12].

It is recommended that these lesions be referred to as focal mucinosis instead of myxoid cysts. There are two reasons for this: first, the lesion is not a true cyst; second, the term “myxoid cyst” could lead to confusion with mucocele, a condition where mucous is retained in the body and true mucous cysts, which are frequently found in the oral cavity [12].

Although the exact etiology of this condition is unknown, according to Johnson and his co-workers there is an overproduction of hyaluronic acid by fibroblasts, which reduces the quantity of collagen produced, makes elastic fibers nearly non-existent, and the collagen fibers that are broken down being replaced by varying amounts of mucin. Tomich noted that the mucoid substance inside the modified connective tissue was alcianophilic at pH 2.5. However, following hyaluronidase treatment, alcianophilia was absent, as hyaluronidase broke down the lesional component, confirming hyaluronic acid to be the responsible factor [12]. According to Neto et al., traumatic stimuli may serve as a predisposing factor for OFM [11]; whereas, Joshi et al. have identified trauma as an important component in increasing the size of these lesions [8].

Systemic diseases associated with mucinosis encompass pretibial myxedema in the presence of hyperthyroidism, myxedema diffusum associated with hypothyroidism, scleroderma, multiple myeloma linked to diabetes, and lichen myxedematosus attributed to diabetes or collagen disorders [3]. Our patient did not have any of these systemic conditions.

The majority of the cases of OFM that have been documented in the English literature have affected women in their fourth or fifth decade of life [13]. Clinically, it manifests as a firm, painless, sessile nodule having the same color as the oral mucosa surrounding it [5]. The gingiva (65.6%) and palate (13.4%) are the most common intraoral locations, although other intraoral sites like buccal mucosa (7.5%), tongue (6.0%), retromolar area (4.5%), and lip (1.5%) may also be involved [3]. Significant radiographic signs are uncommon, though Higuchi et al. reported an intraosseous retromolar growth that was initially misdiagnosed as an odontogenic myxoma before histological confirmation as OFM [3]. An instance of concomitant cervical root resorption in OFM was documented by Gabay et al. [14] while Nilesh et al. reported mild resorption of alveolar bone in their case [6]. It is impossible to diagnose OFM clinically since it mimics a number of other oral lesions having different etiologies, including fibromas, pyogenic granulomas, peripheral ossifying fibromas, peripheral giant cell granulomas, peripheral odontogenic fibromas, and other benign tumors [15].

The diagnosis is based exclusively on histological findings [14] which typically reveal stratified squamous mucosal epithelium with a fairly well-localized area of myxomatous area at the deep and peripheral locations, encircled by a very dense but normal collagenous fibrous connective tissue. The stroma also showed the presence of stellate-shaped fibroblasts adjacent to a few normal collagen fibers [15]. This myxoid stroma could create confusion with other lesions of the head and neck having myxomatous components.

OFM’s key histological differential diagnoses include soft-tissue myxoma, inflammatory fibroepithelial hyperplasia with myxoid degeneration, nerve sheath myxoma, odontogenic myxoma, and mucocele [14].

The myxomas have an infiltrative growth pattern and a thick hyaluronic acid matrix composed of loosely arranged reticulin and collagen fibers, along with small, round, spindle-shaped or stellate fibroblasts. Focal mucinosis lacks the reticulin fiber network and infiltrative development pattern of a true myxoma and only presents with a well-localized myxomatous connective tissue region [12].

Focal mucinosis differs from myxomatous changes in fibrous lesions as the latter merges into the surrounding connective tissue rather than being more clearly delineated. Furthermore, mononuclear inflammatory cells, a strong vascular component, and reticulin fibers are found in areas with myxomatous changes, which cannot be seen in focal mucinosis [12].

The nerve sheath myxoma often has a lobular pattern, with a high concentration of mast cells which is not present in focal mucinosis. The mucous retention cyst (mucocele) is clearly distinguished from focal mucinosis by the presence of a compressed wall of granulation tissue surrounding the accumulated mucus that has extravasated from a lacerated duct. Hyaluronic acid is not found in the mucus-filled area.

Histologically, mucins exhibit blue color when exposed to Alcian Blue at a pH of 2.5. Additionally, mucins can also be stained with mucicarmine, producing a red hue, or with colloidal iron, leading to a blue-green coloration. The specific color observed is contingent upon both the quantity and type of acid groups present in the mucins [16]. The mucicarmine stain is utilized for the detection of mucins originating from epithelial cells. Mucins present in fibroblasts or connective tissues may exhibit limited staining capacity [17]. PAS stain does not stain hyaluronic acid [16]. S-100 staining is negative in OFM while it is positive in myxoid neural lesions such as nerve sheath myxoma [12].

Limited biopsy material from clinicopathologically simulating lesions can yield ambiguities in histopathological diagnosis.

All documented cases of OFM have so far been treated with surgical excision. Only one of these recorded cases has recurred as a result of incomplete resection [18]. However, in most situations, follow-up observations are required.

## Conclusions

Although it is challenging to diagnose the condition based on clinical symptoms and imaging results, OFM should be taken into consideration when diagnosing benign oral tumors, especially involving gingiva. Our view is that in order to validate the clinical suspicion, a focus on histopathologic analysis is important. But the close histological mimics of OFM can range from simple myxomatous changes in benign and or reactive lesions to complex degenerative focal myxomatous changes in high-grade malignant lesions especially sarcomatous tumors like myxoid liposarcoma, myxoid leiomyosarcoma, myxoid chondrosarcoma, and myxofibrosarcoma, etc. hence to rule out such instances of ambiguity, proper differential diagnostic techniques must be applied with meticulous clinicopathological correlation.
